# Supplementary material for: How structural elements evolving from bacterial to human SLC6 transporters enabled new functional properties
Source: BMC Biol. 2018 Mar 14;16:31. doi: 10.1186/s12915-018-0495-6 (PMC5852957; doi:10.1186/s12915-018-0495-6)
Supplement: Supplementary file 1 — Supplementary Figures. Figure S1. Na2 distance time evolution for no-PIP2 system. Figure S2. Na2 distance time evolution for R51W system. Figure S3. Na2 distance time evolution for S/D system. Figure S4. Na2 distance time evolution for K3A/K5A system. Figure S5. Intracellular gating residues distances for S/D system. Figure S6. Intracellular gating residues distances for R51W system. Figure S7. Intracellular gating residues distances for no-PIP2 system. Figure S8. Frequencies of intracellular gating residues interactions. Figure S9. Distribution of the R445–E428 distance in different constructs of hDAT. Figure S10. MSM implied-timescales plots. Figure S11. MSM 1st relaxation mode for no-PIP2 system. Figure S12. Localization density of the N-terminus near the membrane. Figure S13. MSM macrostates for the no-PIP2 systems. (PDF 9.96 mb) [file 12915_2018_495_MOESM1_ESM.pdf]

Additional File 1

How structural elements evolving from bacterial to human SLC6 transporters enabled new functional properties

Asghar M. Razavi[1], George Khelashvili[1,2], Harel Weinstein[1,2]*

[1]Department of Physiology and Biophysics, Weill Cornell Medical College of Cornell University, New York, NY, 10065, USA

[2]Institute for Computational Biomedicine, Weill Cornell Medical College of Cornell University, New York, NY 10065, USA

*Correspondence and requests for materials should be addressed to H.W. (haw2002@med.cornell.edu)

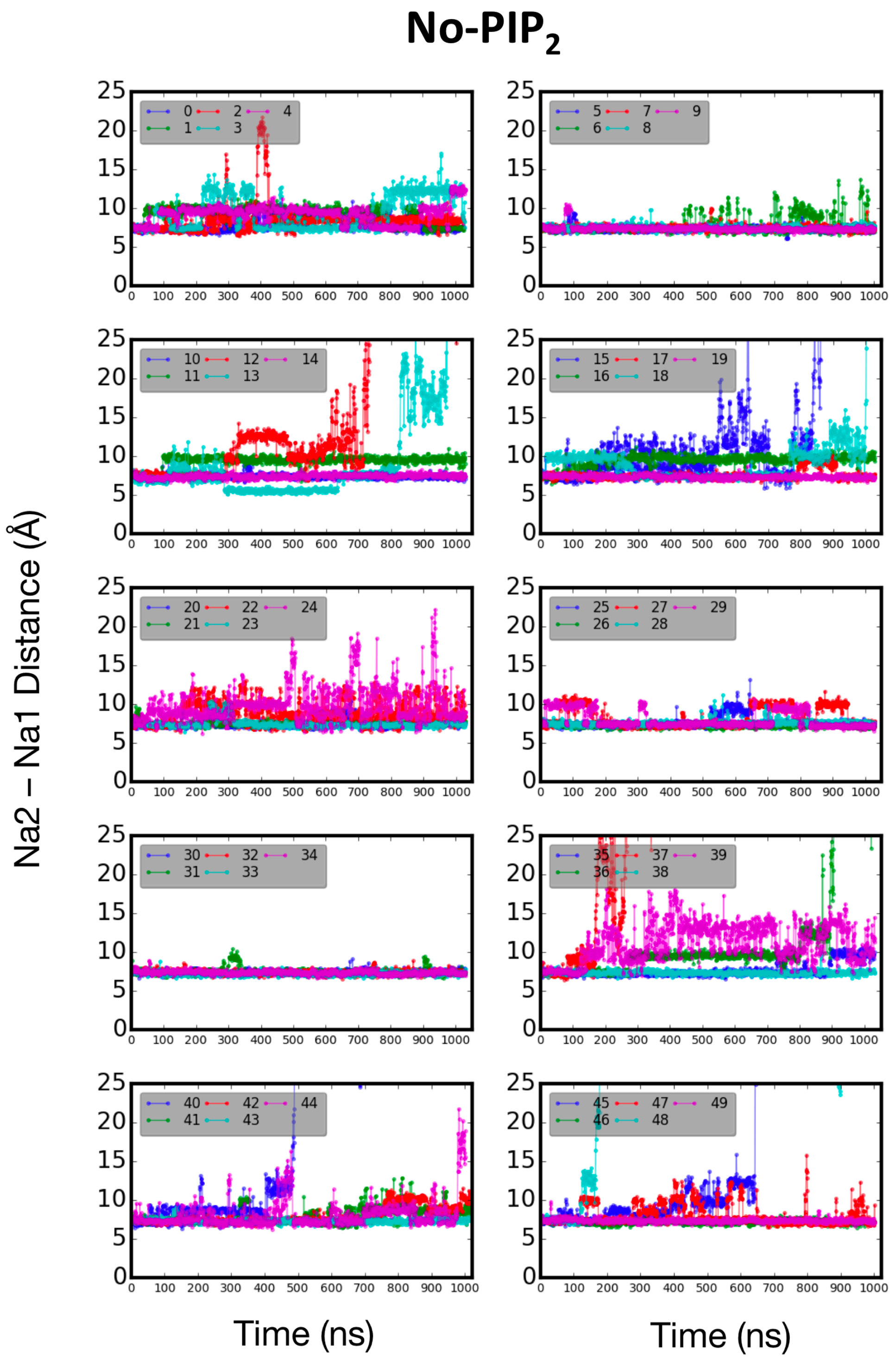

Figure S1. **Na2 distance time evolution for no-PIP$_2$ system**. Time evolution of the distance between sodium at Na2 site (Na+/Na2) and sodium at Na1 site (Na2–Na1) in all 50 MD trajectories of the no-PIP2 system. Na+/Na2 release into intracellular solution (distance beyond 25Å) was observed in 9 trajectories (trajectory numbers 12, 13, 15, 18, 36, 37, 40, 45, and 48).

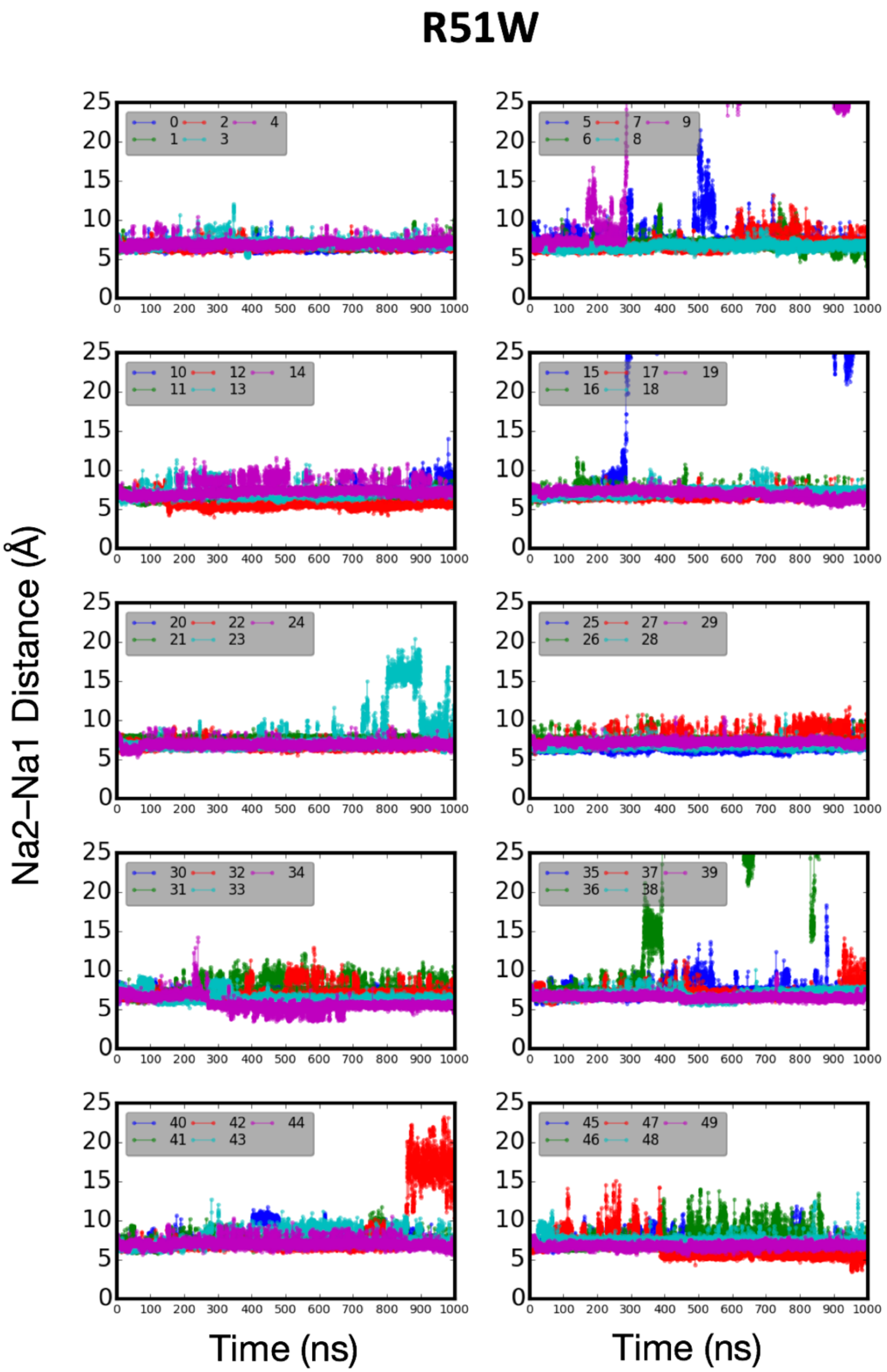

Figure S2. Na2 distance time evolution for R51W system. Same as Figure S1 for the R51W construct.

In trajectories 9, 15, and 36, the Na+/Na2 ion is released to the intracellular environment.

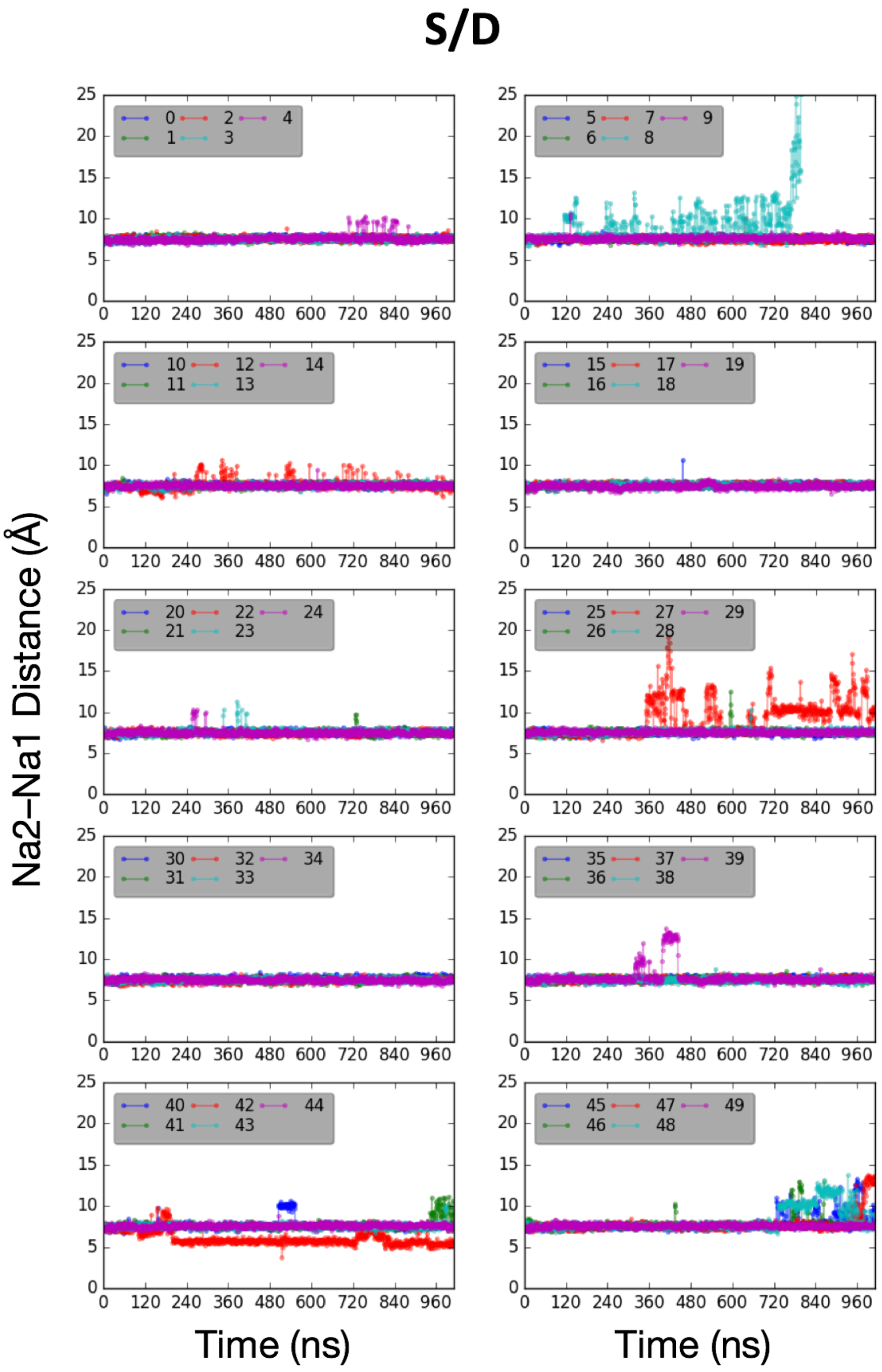

Figure S3. Na2 distance time evolution for S/D system. Same as Figure S1 for the S/D construct. In only one trajectory (# 8) is the Na^+ from Na2 site released to the intracellular environment.

K3A/K5A

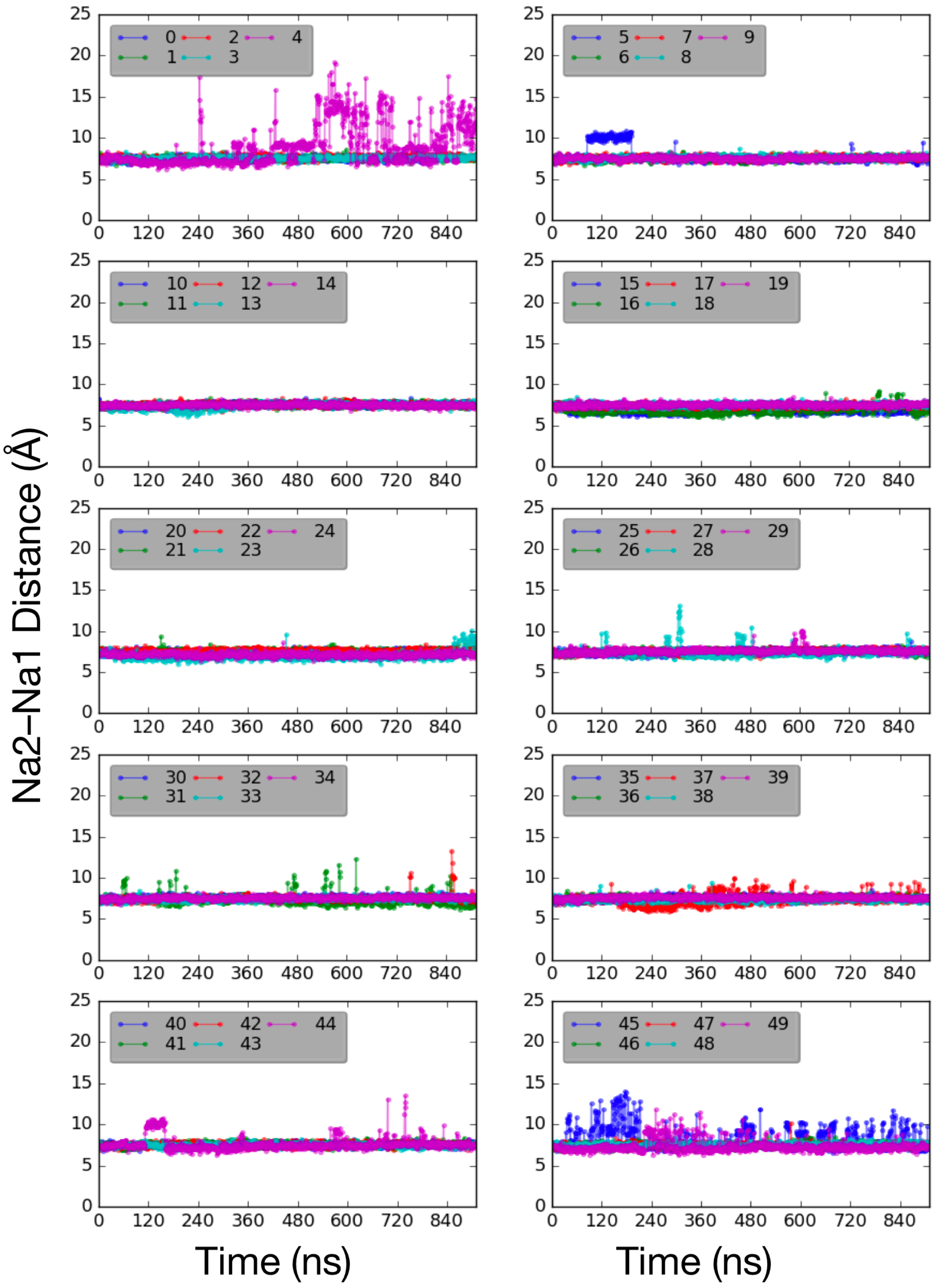

Figure S4. Na2 distance time evolution for K3A/K5A system. Same as Figure S1 for the K3A/K5A construct. No Na+/Na2 release event is observed.

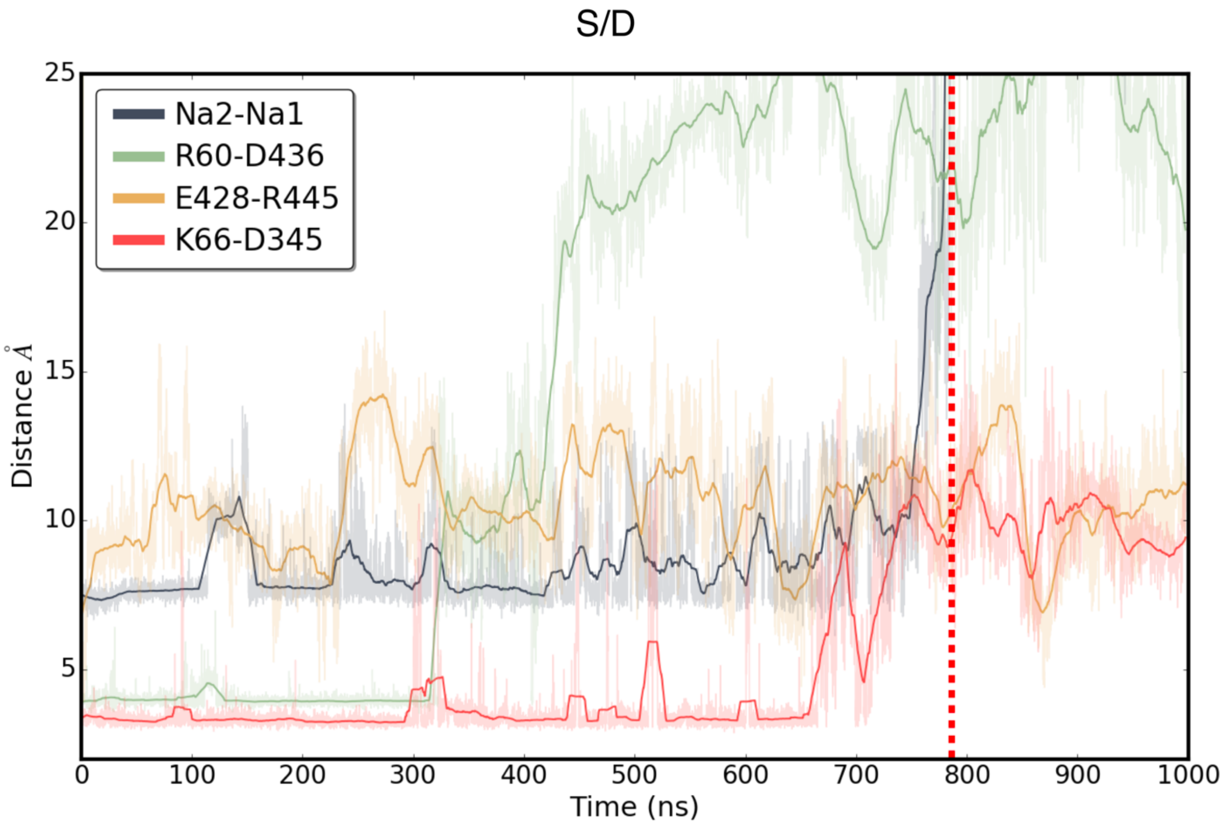

Figure S5. **Intracellular gating residues distances for S/D system**. Time evolution of distances between the residue pairs (R60-D436, E428-R445, K66-D345) that form intracellular gates in trajectory #8 of the S/D construct in which Na+/Na2 is released to the intracellular environment. The time correlation of events is indicated by the Na2–Na1 distance trace. The moving averages are calculated using the adjacent 100 frames. The time of Na+/Na2 is release to the intracellular environment is indicated by the thick dashed red line.

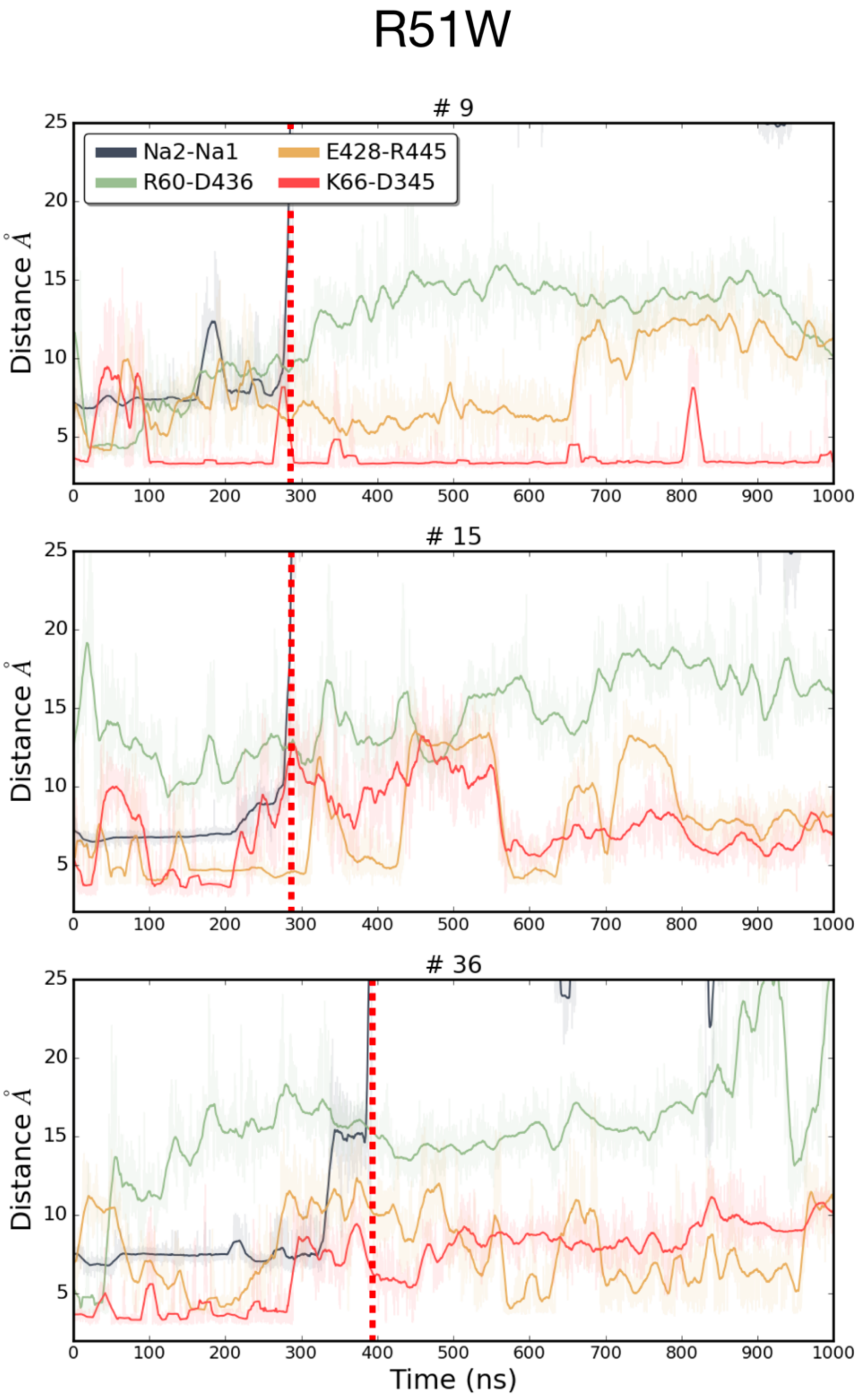

Figure S6. **Intracellular gating residues distances for R51W system**. Same as Figure S5 for the three trajectories in the R51W construct in which Na+/Na2 is released to the intracellular environment.

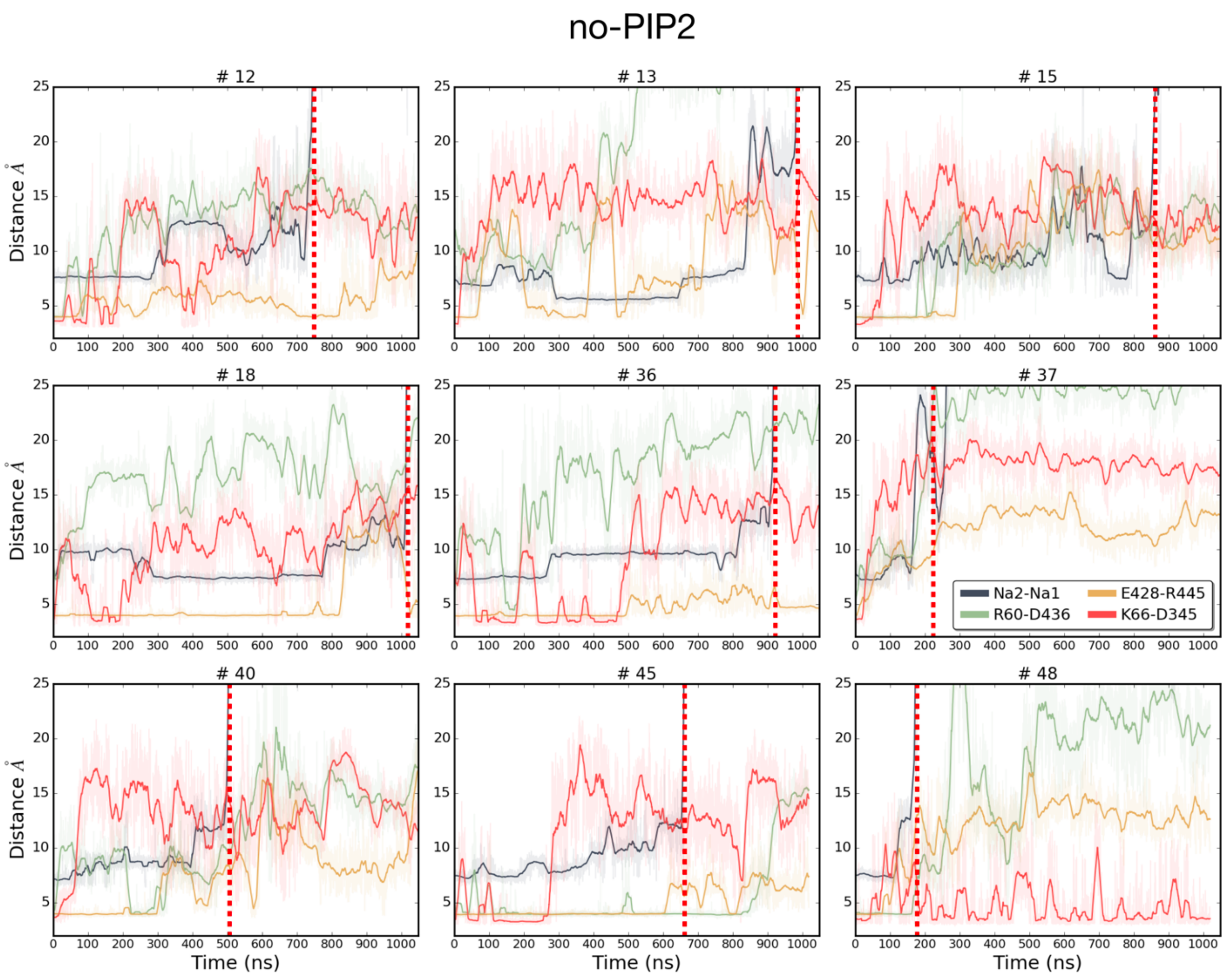

Figure S7. **Intracellular gating residues distances for no-PIP$_2$ system**. Same as Figure S5 for the nine trajectories of the no-PIP2 construct in which Na+/Na2 is released to the intracellular environment.

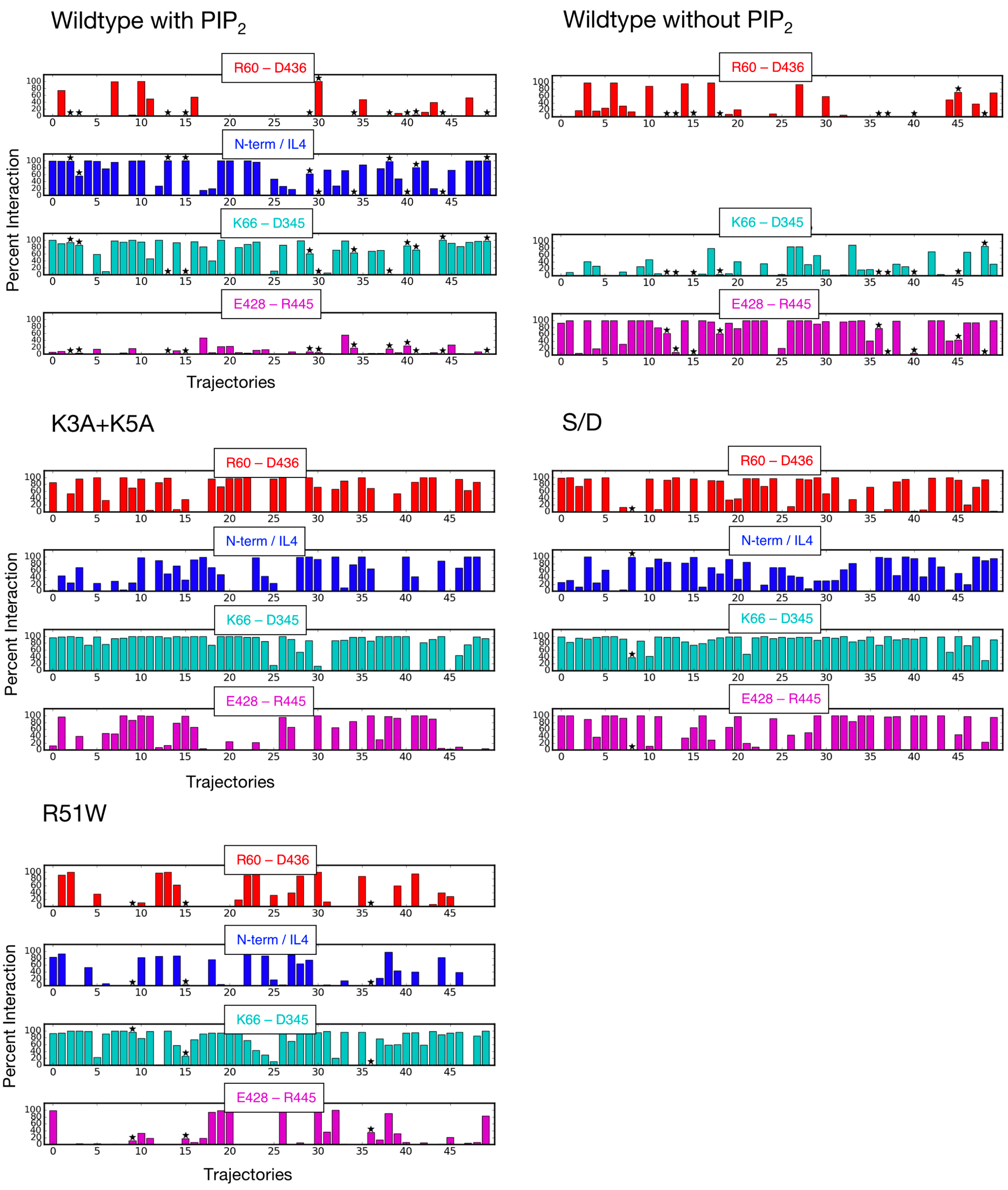

Figure S8. **Frequencies of intracellular gating residues interactions**. The frequencies of interactions between key intracellular gating residues in all 50 trajectories for all systems (expressed as percent), averaged over the simulation time after the firs 500ns, which considered as the equilibration phase. Stars indicate the trajectories in which Na+/Na2 is released. The N-term / IL4 panel represents PIP_2 mediated interactions (within 6 Å) between various Arg and Lys residues from the N-terminus (residue 1 to 59) with Arg443 from IL4 (defined as the interaction of the same PIP_2 with both Arg443 and any Arg or Lys residue of the N-terminus).

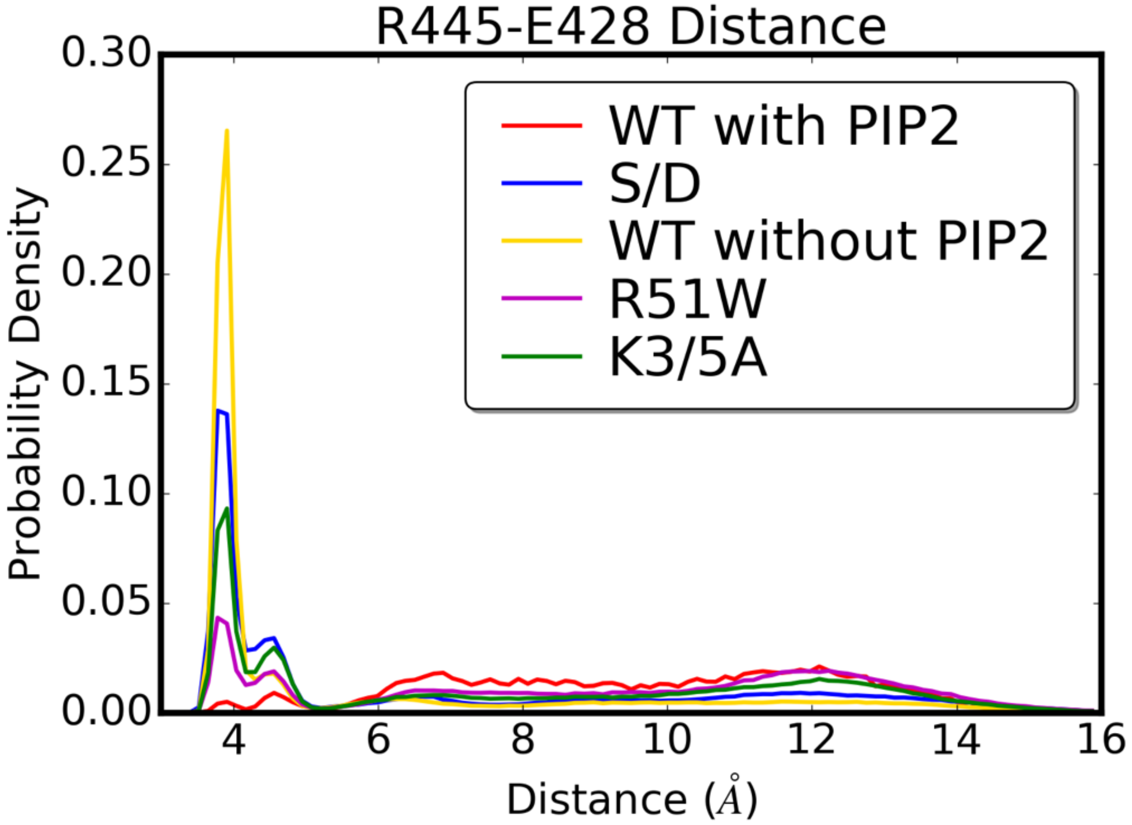

Figure S9. **Distribution of the R445–E428 distance in different constructs of hDAT**. For each construct, all 50 trajectories are used but only the snapshots after the 500 ns in each of them are considered in the calculations.

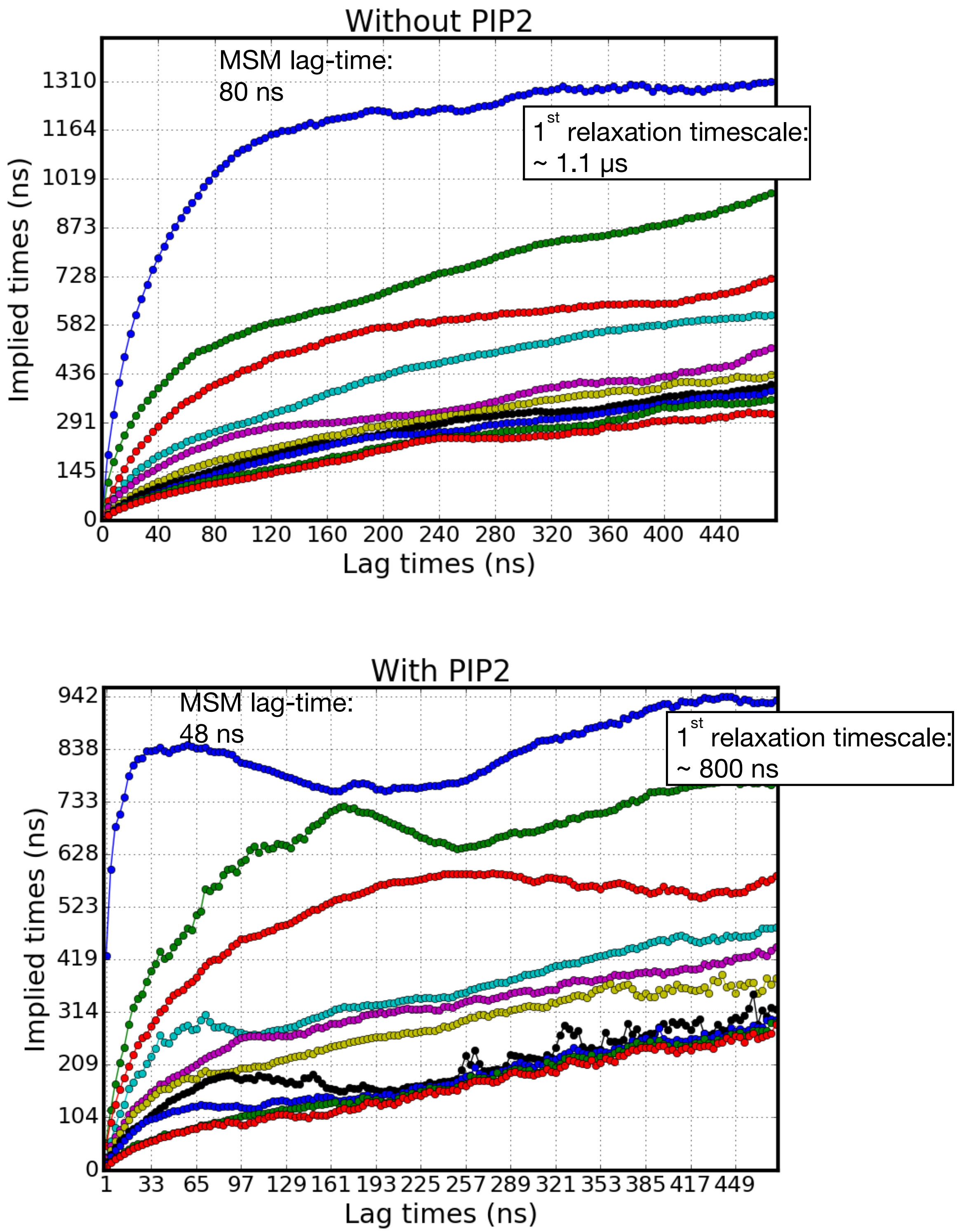

Figure S10. **MSM implied-timescales plots**. Implied time-scales plot obtained from MSM for the no-PIP_2 system (top panel). For comparison, the same plot is shown for the control system in PIP_2-containing membrane from reference 32 (lower panel). The thick red lines highlight the MSM lag-times used for each system to construct the Markovian transition probability matrix. The first MSM relaxation mode for the no-PIP_2 system is on the order of 1.1 μs (corresponding to the Na^+ release from the Na2 site, see Main text), while the same relaxation mode is on the order of ~800 ns for the PIP_2 containing system.

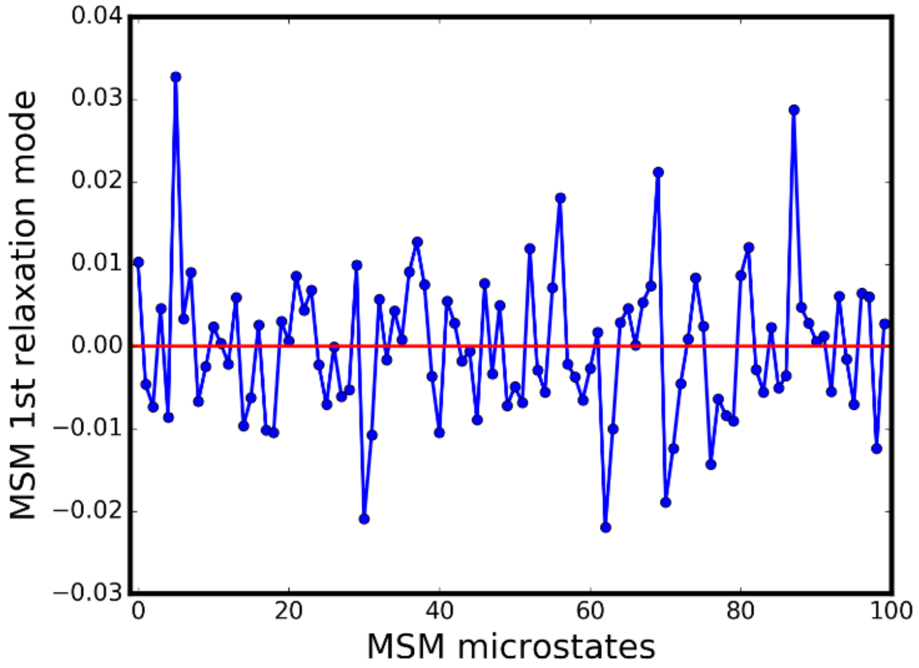

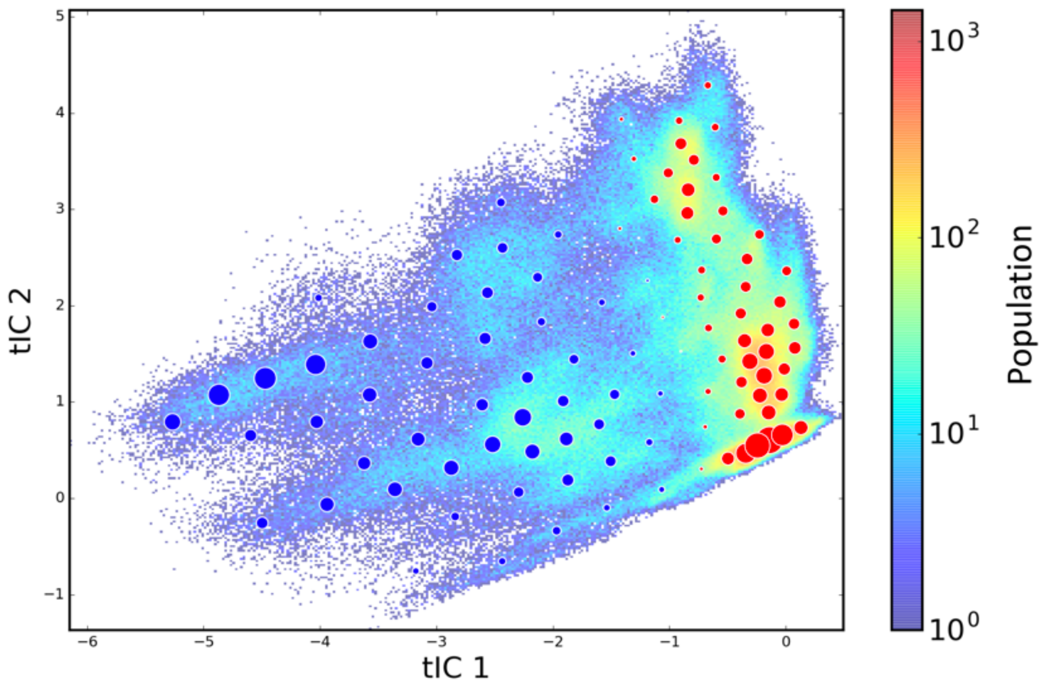

Figure S11. MSM 1st relaxation mode for no-PIP_2 system. (Top) Value of MSM 1st relaxation mode for each microstate in the no-PIP_2 system. The positive valued and negative valued microstates are separated by the red line at zero value. (**Lower**) The location of the 100 microstates (blue and red filled circles) in the 2D tICA landscape showing all snapshots (background colors). The sizes of the filled circles reflect the relative contribution of each microstate to the total population flow from bound Na^+ states to released state.

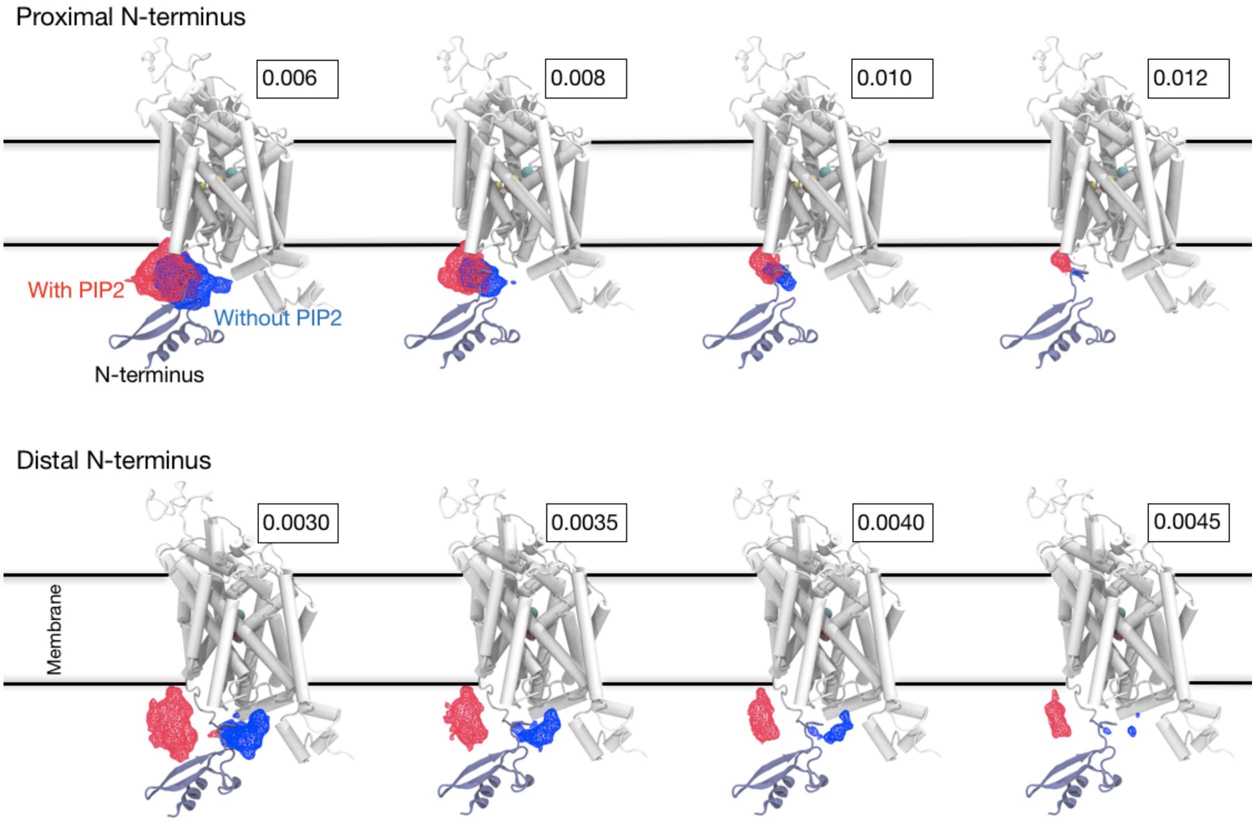

Figure S12. Localization density of the N-terminus near the membrane. (**Top panel**) The localization densities for the proximal part of the N-terminus in the wild-type hDAT for PIP_2-containing trajectories (red mesh) compared to those in the PIP_2-depleted conditions (blue mesh) arranged according to the isovalue (boxed) representing probability of occupancy. (**Lower panel**) Same as the top plot for the distal part of the N-terminus. For each system, a total of 4694 snapshots from all 50 trajectories were selected with a time step of 10 ns between snapshots. Note that for the PIP2-depleted membrane the localization of hDAT N-terminus near membrane surface decreases and the effect is more dramatic for the distal part of the N-terminus.

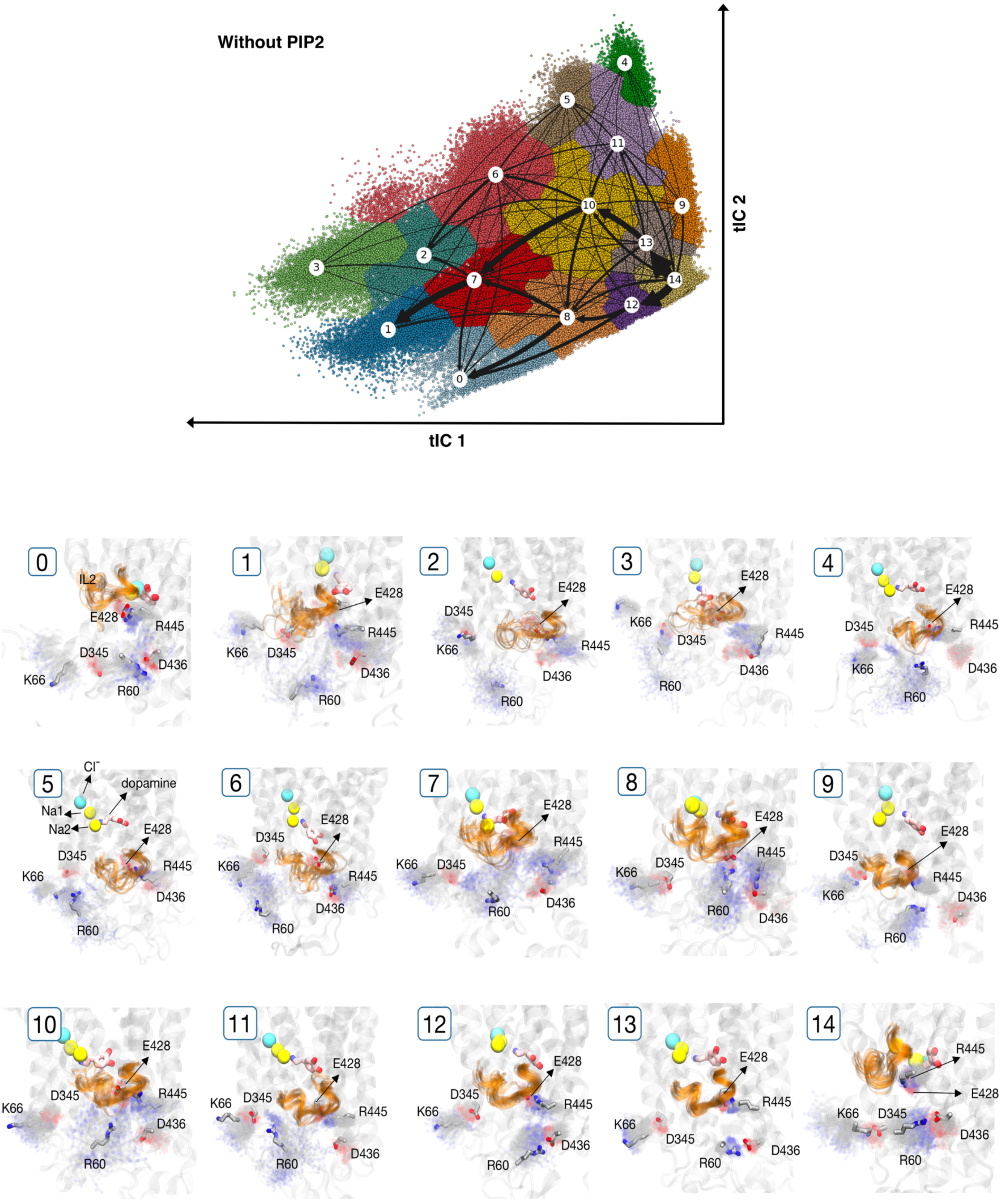
Without PIP2
tIC 1
tIC 2
IL2
E428
R445
D345
D436
K66
R60
Cl⁻
dopamine
Na1
Na2

Figure S13. MSM macrostates for the no-PIP_2 systems. (top) Macrostate representation of the tICA landscape for the wild-type hDAT in PIP_2-depleted membranes. All the pathways for the release of Na+/Na2 from Na+/Na2 bound macrostate (#14) to Na+/Na2 released macrostates (#0, #1, #2, #3) are represented on top of the tICA landscape. Thickness of arrows indicates amount of flux corresponding to pathways (flux values are given in Table S5). (**lower**) Structural representation of each macrostate. Location of the intracellular gate residues (R60, D436, R445, E428, K66, D345) are shown as transparent licorice densities with one representative in solid licorice. The IL2 region is highlighted with orange cartoon representation. Dopamine is shown in pink licorice, sodium ions in yellow spheres, and chloride ion in cyan sphere.
