# Supplementary material for: How structural elements evolving from bacterial to human SLC6 transporters enabled new functional properties
Source: BMC Biol. 2018 Mar 14;16:31. doi: 10.1186/s12915-018-0495-6 (PMC5852957; doi:10.1186/s12915-018-0495-6)
Supplement: Supplementary file 2 — Supplementary Tables. Table S1. Lipid composition of the model membrane. Table S2. Residues included in the calculation of interaction strength between N-terminus and intracellular regions. Table S3. Parameters used for dimensionality reduction and tICA construction. Table S4. Correlation values for water coordination number. Table S5. MSM predicted sodium release pathways. (PDF 169 kb) [file 12915_2018_495_MOESM2_ESM.pdf]

Additional File 2

How structural elements evolving from bacterial to human SLC6 transporters enabled new functional properties

Asghar M. Razavi[1], George Khelashvili[1,2], Harel Weinstein[1,2]*

[1]Department of Physiology and Biophysics, Weill Cornell Medical College of Cornell University, New York, NY, 10065, USA

[2]Institute for Computational Biomedicine, Weill Cornell Medical College of Cornell University, New York, NY 10065, USA

*Correspondence and requests for materials should be addressed to H.W. (haw2002@med.cornell.edu)

Table S1. Lipid composition of the model membrane. The lipid abbreviations are as follows: POPC – 1-palmitoyl-2-oleoyl-sn-glycero-3-phosphocholine, POPE – 1-palmitoyl-2-oleoyl-sn- glycero-3-phosphoethanolamine, POPS – 1-palmitoyl-2-oleoyl-sn-glycero-3-phosphoserine, PI(4,5)P_2- phosphatidylinositol-*4*,*5*-bisphosphate, SM-sphingomyelin.

Lipid	Extracellular	Intracellular	Total
**Cholesterol**	29	24	53
**POPC**	125	26	151
**POPE**	0	92	92
**POPS**	0	20	20
**PI(4,5)P_2**	0	18	18
**SM**	12	0	12
**Number of lipids**	166	180	346

Table S2. Residues included in the calculation of interaction strength between N-terminus and intracellular regions.

Domain	Selected Residues
Distal N-terminus	K3, K5, K19, E20
Proximal N-terminus	K27, E28, E30, K35, E36, R51, E56
IL1	R125, E126, K133, K139
IL2	K257, K260, K264
IL3	K337, R344
IL4	R443, R445, E446
IL5	D506, D507, R515, R521, K525
C-terminus	K579, R588, E589, K590, E598, K599, D600, R601, E602, D605, R606, E608, R610, R615, K619

Table S3. Parameters used for dimensionality reduction and tICA construction.

Parameters reflecting Na^+ motion	Parameters related to water penetration
Na+/Na2 to Na+/Na1 distance	R60–Y335 distance
Na+/Na2 to E428 distance	R60–E446 distance
Na+/Na2 to D421 distance	R60–E428 distance
Na+/Na2 to D79 distance	R60-D436 distance
	Y335–E428 distance
	D436–R445 distance
	E428–R445 distance
	Na+/Na2 water coordination number

Table S4. Correlation values for water coordination number of Na^+ from Na2 site and the release process, measured as distance between this sodium from the sodium at Na1. The correlation numbers are calculated by considering, in the trajectory of each construct, all the frames up to the time when Na^+ from Na2 site has reached the intracellular environment. For comparison, values for the wild type system are also listed (reference 32).

**Phenotype**	**Correlation values**											
**R51W**	0.60	0.61	0.74									
**S/D**	0.54											
**No-PIP2**	0.75	0.58	0.51	0.86	0.80	0.56	0.60	0.75	0.82			
**WT***	0.76	0.70	0.56	0.38	0.41	0.74	0.60	0.75	0.78	0.50	0.62	0.44

*These values are taken from reference 32.

Table S5. **MSM predicted sodium release pathways and fluxes for the wild-type hDAT in PIP2-containing and PIP2-depleted membranes.** The pathways highlighted in red, green, and blue in Figure 4 (main text) are the three major flux paths discussed in the main text.

Without PIP2				With PIP2		
Path	Flux	Cumulative flux		Path	Flux	Cumulative flux
[14 13 10 7 1]	0.217	0.217		[14 12 7]	0.197	0.197
[14 12 8 0]	0.141	0.358		[14 13 7]	0.094	0.292
[14 12 0]	0.113	0.471		[14 13 11 4 1]	0.079	0.371
[14 10 8 7 0]	0.082	0.553		[14 7]	0.075	0.446
[14 13 11 10 6 2]	0.065	0.617		[14 13 3]	0.074	0.520
[14 13 8 1]	0.053	0.671		[14 11 3]	0.056	0.577
[14 8 7 2]	0.046	0.717		[14 10 8 5]	0.051	0.628
[14 13 7 1]	0.033	0.75		[14 10 5]	0.038	0.665
[14 12 10 2]	0.03	0.78		[14 10 12 3]	0.037	0.702
[14 11 5 6 3]	0.03	0.809		[14 13 4 3]	0.035	0.737
[14 9 11 6 2]	0.027	0.836		[14 13 2 3]	0.033	0.770
[14 9 10 2]	0.018	0.854		[14 10 12 7]	0.031	0.802
[14 8 0]	0.016	0.87		[14 11 9 4 7]	0.016	0.818
[14 13 6 0]	0.016	0.887		[14 8 5]	0.016	0.833
[14 7 2]	0.016	0.903		[14 5]	0.014	0.847
[14 11 10 6 7 3]	0.015	0.917		[14 13 11 7]	0.013	0.861
[14 13 8 2]	0.014	0.932		[14 13 9 3]	0.013	0.874
[14 0]	0.012	0.944		[14 2 7]	0.013	0.887
[14 12 7 2]	0.011	0.955		[14 9 6 4 1]	0.011	0.897
[14 13 9 10 8 6 0]	0.006	0.961		[14 3]	0.011	0.908
[14 11 7 3]	0.005	0.966		[14 13 1]	0.010	0.918
[14 11 5 6 1]	0.004	0.971		[14 11 1]	0.010	0.928
[14 10 7 1]	0.004	0.975		[14 8 7]	0.009	0.937
[14 13 4 10 0]	0.003	0.977		[14 10 7]	0.007	0.944
[14 12 11 6 0]	0.003	0.98		[14 13 12 5]	0.007	0.951
[14 12 13 8 0]	0.002	0.982		[14 10 13 4 5]	0.006	0.956
[14 6 1]	0.002	0.985		[14 13 2 7]	0.006	0.962
[14 9 7 3]	0.002	0.987		[14 10 13 12 7]	0.005	0.967
[14 12 1]	0.002	0.988		[14 4 1]	0.005	0.972
[14 12 6 1]	0.001	0.99		[14 13 12 4 2 7]	0.004	0.976
[14 11 10 6 1]	0.001	0.991		[14 10 13 11 1]	0.004	0.980
[14 9 10 1]	0.001	0.993		[14 10 13 12 1]	0.003	0.983
[14 5 6 2]	0.001	0.994		[14 13 9 1]	0.003	0.986
[14 9 4 11 8 3]	0.001	0.995		[14 10 13 12 2 1]	0.003	0.989
[14 12 7 3]	0.001	0.996		[14 10 11 2 1]	0.003	0.992
Total flux for major pathway 1:		0.32		Total flux for major pathway 1:		0.48
Total flux for major pathway 2:		0.28		Total flux for major pathway 2:		0.39
Total flux for major pathway 3:		0.40		Total flux for major pathway 3:		0.13
